# Development and application of multiplex PCR method for simultaneous detection of seven viruses in ducks

**DOI:** 10.1186/s12917-019-1820-1

**Published:** 2019-04-01

**Authors:** Ming Yao, Xiyu Zhang, Yunfei Gao, Suquan Song, Danning Xu, Liping Yan

**Affiliations:** 1 MOE Joint International Research Laboratory of Animal Health and Food Safety, College of Veterinary Medicine, Nanjing Agricultural University, Nanjing, 210095 China; 2 Guangdong Province Key Laboratory of Waterfowl Healthy Breeding, Zhongkai University of Agricultural and Engineering, Guangzhou, 510225 China; 3 Nanjing Tianbang Bio-Industry co., LTD, Nanjing, 211102 China; 4 Jiangsu Detection Center of Terrestrial Wildlife Disease, Nanjing Agricultural University, Nanjing, 210095 People’s Republic of China

**Keywords:** M-PCR, Duck, Virus, Clinical detection

## Abstract

**Background:**

Major viruses, including duck-origin avian influenza virus, duck-origin Newcastle disease virus, novel duck parvovirus, duck hepatitis A virus, duck Tembusu virus, fowl adenovirus, and duck enteritis virus, pose great harm to ducks and cause enormous economic losses to duck industry. This study aims to establish a multiplex polymerase chain reaction (m-PCR) method for simultaneous detection of these seven viruses.

**Results:**

Specific primers were designed and synthesized according to the conserved region of seven viral gene sequences. Then, seven recombinant plasmids, as the positive controls, were reconstructed in this study. Within the study, D-optimal design was adopted to optimize PCR parameters. The optimum parameters for m-PCR were annealing temperature at 57 °C, Mg^2+^ concentration at 4 mM, *Taq* DNA polymerase concentration at 0.05 U/μL, and dNTP concentration at 0.32 mM. With these optimal parameters, the m-PCR method produced neither cross-reactions among these seven viruses nor nonspecific reactions with other common waterfowl pathogens. The detection limit of m-PCR for each virus was 1 × 10^4^ viral DNA copies/μL. In addition, the m-PCR method could detect a combination of several random viruses in co-infection analysis. Finally, the m-PCR method was successfully applied to clinical samples, and the detection results were consistent with uniplex PCR.

**Conclusion:**

Given its rapidity, specificity, sensitivity, and convenience, the established m-PCR method is feasible for simultaneous detection of seven duck-infecting viruses and can be applied to clinical diagnosis of viral infection in ducks.

**Electronic supplementary material:**

The online version of this article (10.1186/s12917-019-1820-1) contains supplementary material, which is available to authorized users.

## Background

The Food and Agriculture Organization (FAO) suggests that approximately 818 million ducks were raised annually in China, accounting for 65.96% of the World’s stock in 2016 (http://www.fao.org/faostat/en/#data/QA). However, along with the development of China’s duck industry, many problems emerged. Among them, viral infection is one of the most important problems endangering the waterfowls [1]. The major viruses that cause enormous economic losses to duck industry include duck-origin avian influenza virus (AIV), duck-origin Newcastle disease virus (NDV), novel duck parvovirus (NDPV), duck hepatitis A virus (DHAV), duck Tembusu virus (DTMUV), fowl adenovirus (FAdV), and duck enteritis virus (DEV) [2].

AIV has been isolated from numerous avian species. Normally, waterfowls are the primary reservoir hosts. However, highly pathogenic avian influenza could cause high mortality in ducks and geese [3]. Therefore, determining AIV in ducks is important in epidemiology research [4]. Similar to AIV, waterfowls are generally considered potential reservoirs for NDV, which has been occasionally reported in China in ducks. In addition, since 1997, Newcastle disease occurred frequently in geese throughout China, causing devastating economic losses [5]. NDPV, a new variant of goose parvovirus, is a novel duck parvovirus. The virus is currently infecting ducks across China, the morbidity of which is approximately 20–40%, thereby causing serious economic loss for the duck industry in China [6]. DHAV is a highly contagious, acute, fatal disease of ducklings, causing more than 80% of mortality in ducklings under three weeks old. Therefore, DHAV has jeopardized the duck industry throughout the world [7, 8]. DTMUV is another newly emerged infectious disease mainly affecting laying ducks. When the ducklings are infected with DTMUV, the morbidity can reach 90%, and the mortality rate can vary from 5 to 30% [9]. FAdV is another major concern in the poultry industry; it may cause immunodeficiency or vaccination failure and result in serious economic loss [10]. DEV could also cause considerable economic losses to the commercial duck industry due to the high mortality and decreased egg production rate. Therefore, it poses a continuous threat to wild and migratory waterfowl populations [11].

The development of rapid and convenient methods to determine these viruses and accordingly implementing preventive measures to reduce economic losses as soon as possible [1, 12] are important given the great danger of these viruses on the duck industry. Thus far, many methods, such as virus isolation and identification, serological detection, immuno-electron microscopy, enzyme-linked immune sorbent assay (ELISA), lateral flow assay (LFA), and Polymerase Chain Reaction (PCR) techniques, have been applied for virus detection [13–18]. Virus isolation and identification is a confirmation method for virus detection. However, the method is time consuming, greatly hindering its application in rapid clinical detection [19]. Immunoassay-based methods, such as ELISA, have been widely used. The problem of such technique is the requirements of special antibodies, whose production is time consuming and exhausting [20]. Immuno-electron microscopy requires sophisticated instrumentation and high amount of virus; thus, it is not feasible in clinical diagnosis [21]. Different from these methods, PCR is a common used technique in molecular biology [22]. It can exponentially amplify a single copy or few copies of a specific DNA segment. It has been widely used in clinical laboratory research for a broad variety of pathogen detection because of its high sensitivity, non-strict detection conditions, strong specificity, high speed, and safety [20, 23].

The m-PCR refers to the PCR reactions, in which two or more primer pairs are used in a PCR reaction tube, and multiple nucleic acid fragments are amplified simultaneously [24]. Compared with uniplex PCR, m-PCR shows unparalleled advantages, including high amplification efficiency, time-saving, and high throughput [25, 26]. More importantly, this approach can differentially diagnose various viruses at the same time; it is an effective method for rapid diagnosis of mixed-virus infection in clinical detection [19, 27]. We aimed to develop and optimize a single-step m-PCR method capable of detecting and differentiating seven major duck viruses, including AIV, NDV, NDPV, DHAV, DTMUV, FAdV, and DEV.

## Results

### Optimization and establishment of the m-PCR method

**Fig. 1:**
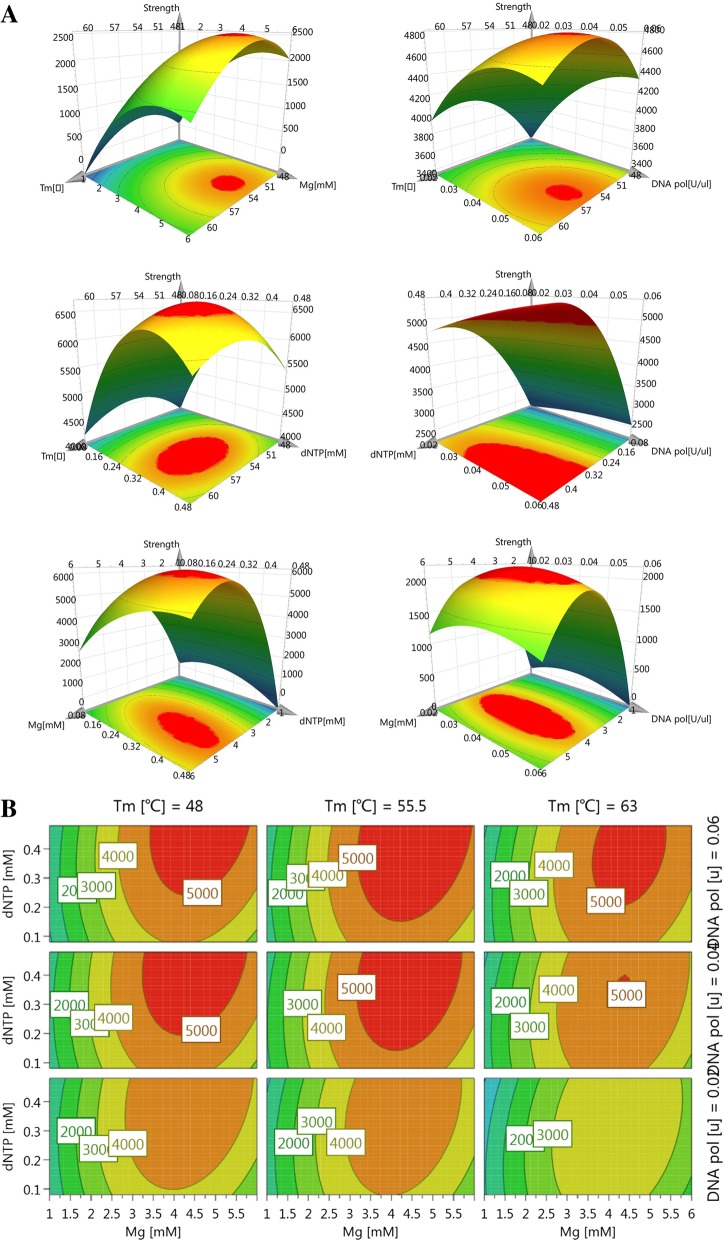
Response surface plots for DHAV. Different response plots for DHAV for various combinations of the investigated parameters. **a**: Different combinations of four factors to form the three-dimension response surface curves; Mg: Mg^2+^ concentration, Tm: the annealing temperature, DNA pol: Taq DNA polymerase concentration, dNTP: dNTP concentration. **b**: 4D plots indicated the interaction between the four factors

**Fig. 2:**
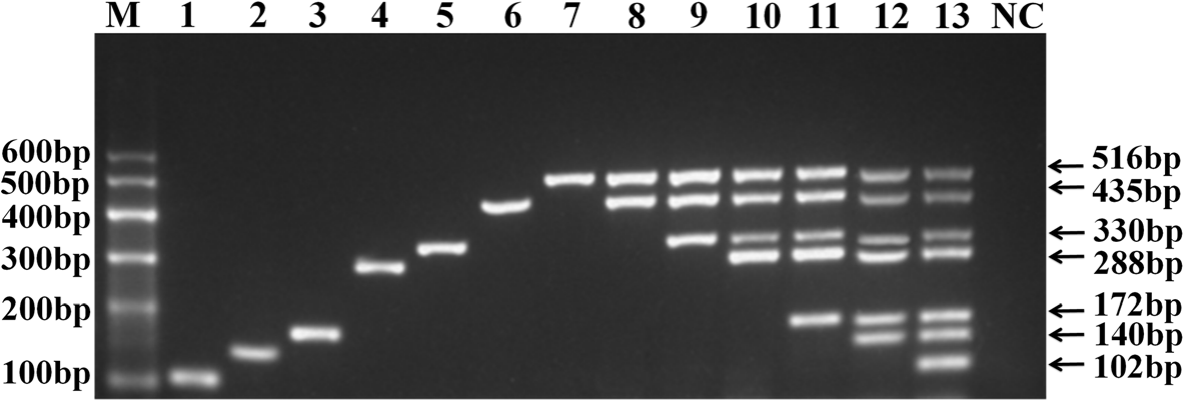
Agarose gel electrophoresis of the uniplex and m-PCR products from single or multiple combinations of viruses. 1, FAdV (102 bp); 2, DHAV (140 bp); 3, DEV (172 bp); 4, DTMUV (288 bp); 5, NDV (330 bp); 6, AIV (435 bp); 7, NDPV (516 bp). 8, NDPV+AIV; 9, NDPV+AIV + NDV; 10, NDPV+AIV + NDV + DTMUV; 11, NDPV+AIV + NDV + DTMUV+DEV; 12, NDPV+AIV + NDV + DTMUV+DEV + DHAV; 13, NDPV+AIV + NDV + DTMUV+DEV + DHAV+FAdV; NC, negative control

A D-optimal design was used to optimize the m-PCR method with 22 runs performed in one randomized batch (in duplicate measurements). As an example, the three-dimension response surface curves of DHAV are shown in Fig. 1a. Similarly, 4D plots indicated the interaction between the four factors (Fig. 1b). We obtained the ultimate optimum parameters, considering the economic perspective, with the necessary compromise, with annealing temperature at 57 °C, Mg^2+^ concentration at 4 mM, *Taq* DNA Polymerase concentration at 0.05 U/μL, and dNTP concentration at 0.32 mM. With the ultimate optimum primers and parameters (Table 1), we successfully established the m-PCR method, which could also effectively amplify genes of duplex, triplex, and even septuplet (Fig. 2).

**Table 1:** Primers used in this study

Viruses	Primer sequences (5′-3′)	Targeted genes	Position	Accession no.	Product size (bp)	Working concentration (μM)
FAdV	F: CAACAGCCTCTCGTACCCAG	Hexon	839–858	AJ459805	102	0.12
	R: CCGATGTAGTTGGGCCTGAG		921–940			
DHAV	F: CTTTCCACTCCCTGCTCCC	VP1	510–528	KJ606043	140	0.12
	R: TTGGCTTCCACATCCTCTTCA		629–649			
DEV	F: ATCGCATGTAGACGTTGGTT	UL2	726–745	EU885419	172	0.16
	R: AGACAGCGGTGATGGATGG		879–897			
DTMUV	F: AATCGGTAGTGGCTTTGG	Envelope	432–449	AB110495	288	0.28
	R: AGTCTGCCGACATGGATAT		701–719			
NDV	F: CACCGGCAACCCTATTCTGT	Fusion	853–872	M24701	330	0.28
	R: AGTGCGCCTTCAGTCTTTGA		1163–1182			
AIV	F: GGCGACTACTACCAACCCA	Matrix	521–539	DQ064401	435	0.24
	R: CTGCTGTTCCTGCCGATAT		937–955			
NDPV	F: TATGTCCTGGGCTCGGCTAC	VP3	448–467	EF014903	516	0.24
	R: AGCTGACACAGGTCCAGGTT		944–963

### Specificity of the m-PCR method

**Fig. 3:**
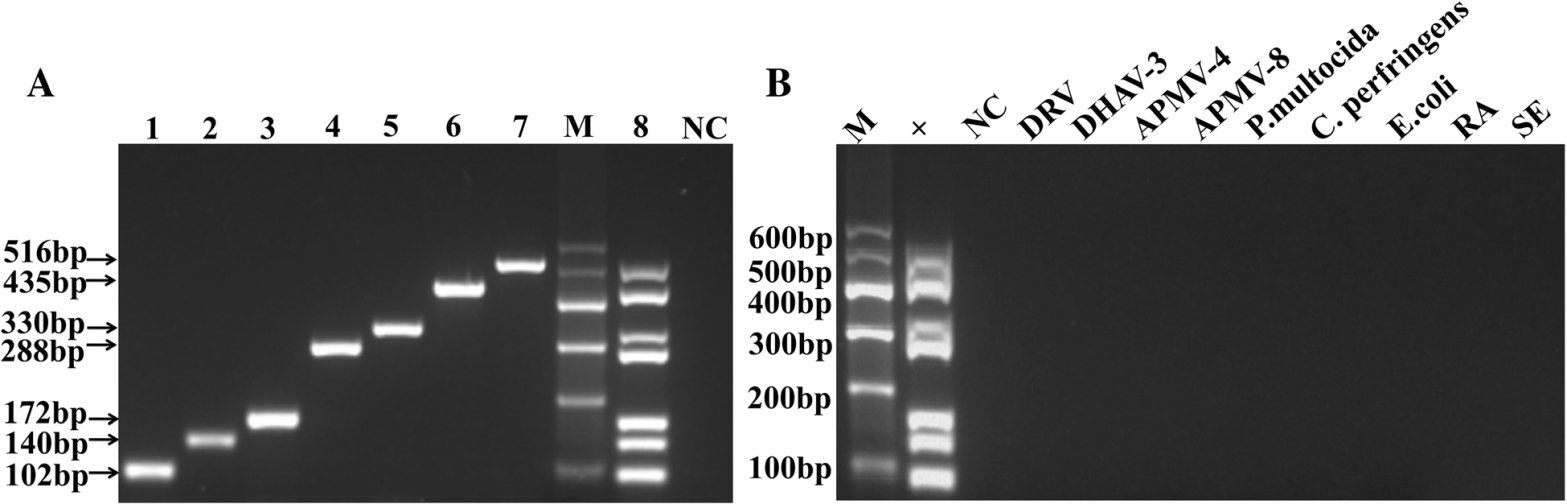
Specificity of the m-PCR method. **a**: Lanes 1–7, seven virus templates were detected (102 bp for FAdV, 140 bp for DHAV, 172 bp for DEV, 288 bp for DTMUV, 330 bp for NDV, 435 bp for AIV, and 516 bp for NDPV); Lane 8, result of the m-PCR method (mixture of the same concentrations of seven viruses); M, DL600 marker; NC, negative control. **b**: Specificity of the m-PCR method with other pathogens; 1 (M), DL600 marker; 2 (+), positive control; 3 (NC), negative control; 4 (DRV), duck Reovirus; 5 (DHAV-3), DHAV serotype 3; 6 (APMV-4), Avian paramyxoviruses serotype 4; 7 (APMV-8), Avian paramyxoviruses serotype 8;8 (*P. multocida*), *Pasteurella multocida;*9 (*C. perfringens*), *Clostridium perfringens*; 10 (*E. coli*), *Escherichia coli*; 11 (RA), *Riemerella anatipestifer*; 12 (SE), *Salmonella*

The specificity of the m-PCR method was evaluated with these seven viruses and DRV, APMV-4, APMV-8, DHAV-3, *Escherichia coli*, *Salmonella*, *Riemerella anatipestifer*, *Pasteurella multocida*, and *Clostridium perfringens* that may infect ducks. As shown in Fig. 3a, the band for each virus was clear for m-PCR analysis, similar to that of uniplex PCR. Furthermore, although other viruses or bacteria genomes were mixed in the sample pool, only the genes of these seven viruses were specifically amplified; no amplification occurred with those interfering genomes (Fig. 3b). The sequencing results further demonstrated the good specificity of the m-PCR.

### Sensitivity of the m-PCR method

**Fig. 4:**
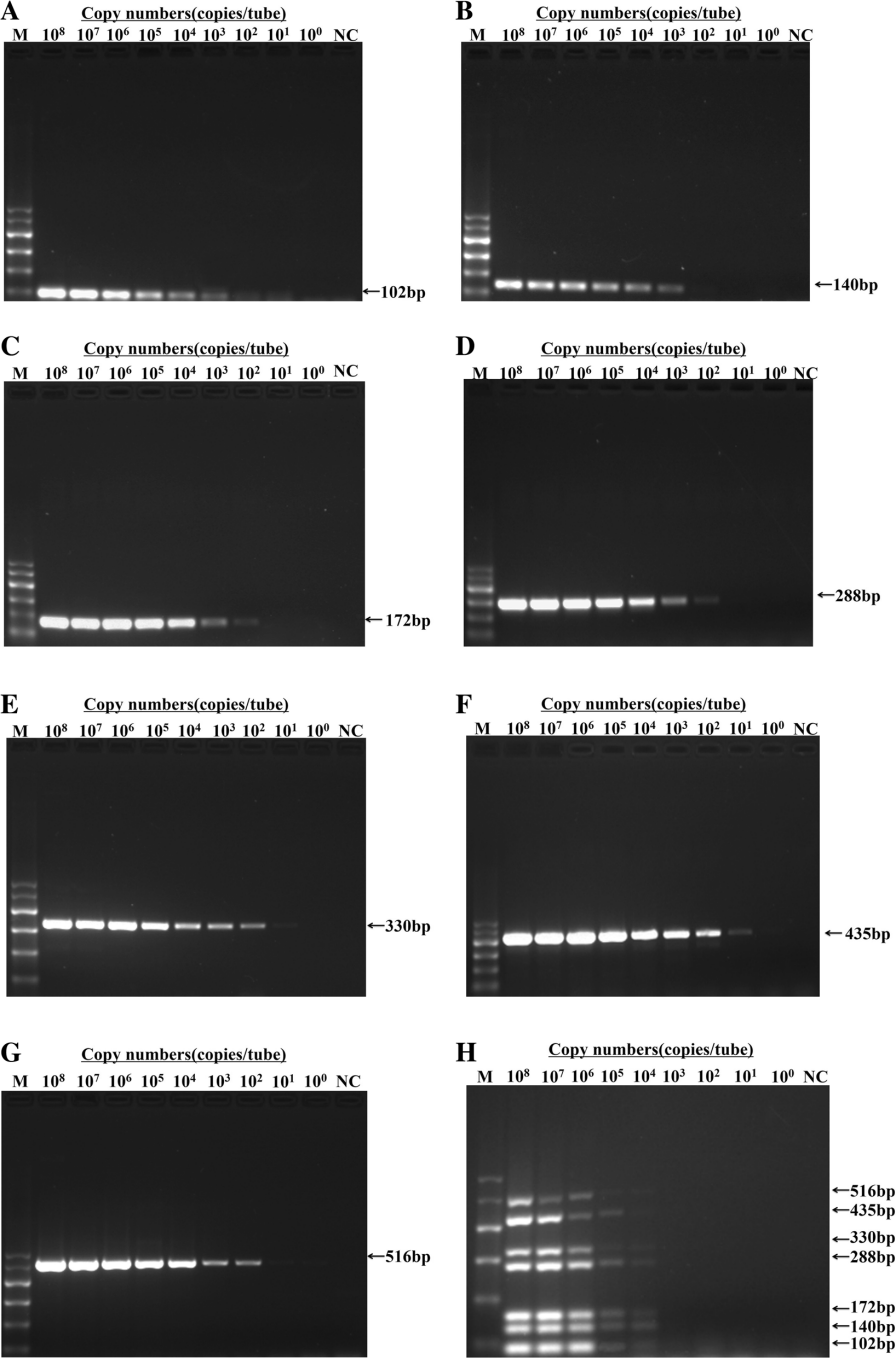
Sensitivity of the m-PCR method. Templates of pMD-FAdV (**a**), pMD-DHAV (**b**), pMD-DEV (**c**), pMD-DTMUV (**d**), pMD-NDV (**e**), pMD-AIV (**f**), pMD-NDPV (**g**), and mixture of seven virus plasmids (**h**). M, DL600 marker; NC, negative control. Plasmids were diluted from 10^8^ to 10^0^ DNA copies/μL

Each of the seven recombinant plasmids and the mixed plasmids were diluted from 1 × 10^8^ to 1 × 10^0^ copies/μL by 10-fold gradient dilution. Then, m-PCR was performed. As shown in Fig. 4, when single template was applied, the detection limits were 1 × 10^2^ copies/μL for DEV, DTMUV, and NDPV, 1 × 10^3^ copies/μL for FAdV and DHAV, and 1 × 10^1^ copies/μL for NDV and AIV. When seven DNA templates were mixed, the detection limit of each virus was 1 × 10^4^ copies/μL.

### Reproducibility of the m-PCR method

The results of reproducibility of the uniplex PCR and m-PCR method are shown in Additional file 1: Figure S1. Uniplex PCR was conducted with plasmids of concentrations of 10^2^, 10^3^, 10^4^, and 10^5^ copies/μL and 10^4^, 10^5^, and 10^6^ copies/μL for m-PCR method, confirming that the proposed method was reliable.

### Co-infection model and clinical sample detection

**Fig. 5:**
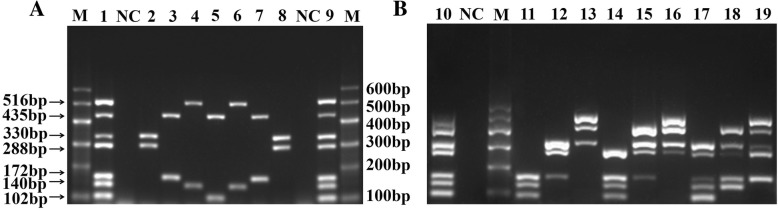
Co-infection analysis with m-PCR method. (**a**): Detection result of co-infection of two viruses. 1 and 9, positive control; 2 and 8, NDV + DTMUV; 3 and 7, AIV + DEV; 4 and 6, DHAV+NDPV; 5, AIV + FAdV; (**b**): Detection result of co-infection of several viruses. 10, positive control; 11, DEV:DHAV:FAdV = 10^7^:10^6^:10^5^; 12, NDV:DTMUV:DEV = 10^7^:10^6^:10^5^; 13, NDPV:AIV:NDV = 10^7^:10^6^:10^5^; 14, DTMUV:DEV:DHAV:FAdV = 10^8^:10^7^:10^6^:10^5^; 15, AIV;NDV:DTMUV:DEV = 10^8^:10^7^:10^6^:10^5^; 16, NDPV:AIV:NDV:DTMUV = 10^8^:10^7^:10^6^:10^5^; 17, NDV:DTMUV:DEV:DHAV:FAdV = 10^7^:10^6^:10^5^:10^6^:10^7^; 18, AIV;NDV:DTMUV:DEV:DHAV = 10^7^:10^6^:10^5^:10^6^:10^7^; 19, NDPV:AIV:NDV:DTMUV:DEV = 10^7^:10^6^:10^5^:10^6^:10^7^; NC, negative control; M, DL600 marker

As shown in Fig. 5a and Additional file 2: Figure S2, we simulated duplex infections of different combinations of viruses in the same concentration. Simultaneously, triplex, quadruple, and quintuple infections of different combinations of viruses in the different concentrations were detected (Fig. 5b). In addition, a total of 60 clinical samples were examined using the developed m-PCR method and uniplex PCR. The result showed that 11 samples were virus positive, including 1 positive sample for FAdV, 4 positive samples for DHAV, 2 positive samples for DEV, 2 positive samples for DTMUV, 2 positive samples for AIV, and 1 positive sample for NDPV. The positive rate was 18%. Among these 11 positive samples, sample 8 was co-infected with DHAV and AIV (Table 2). Moreover, these results were further confirmed by the primers published previously.

**Table 2:** Result of the clinical positive samples detected by m-PCR and uniplex PCR

Positive sample	Viruses						
	FAdV	DHAV	DEV	DTMUV	NDV	AIV	NDPV
1	-(−)	+(+)	-(−)	-(−)	-(−)	-(−)	-(−)
2	-(−)	-(−)	-(−)	+(+)	-(−)	-(−)	-(−)
3	-(−)	-(−)	+(+)	-(−)	-(−)	-(−)	-(−)
4	-(−)	-(−)	-(−)	-(−)	-(−)	+(+)	-(−)
5	-(−)	-(−)	-(−)	-(−)	-(−)	-(−)	+(+)
6	-(−)	+(+)	-(−)	-(−)	-(−)	-(−)	-(−)
7	+(+)	-(−)	-(−)	-(−)	-(−)	-(−)	-(−)
8	-(−)	+(+)	-(−)	-(−)	-(−)	+(+)	-(−)
9	-(−)	-(−)	-(−)	+(+)	-(−)	-(−)	-(−)
10	-(−)	+(+)	-(−)	-(−)	-(−)	-(−)	-(−)
11	-(−)	-(−)	+(+)	-(−)	-(−)	-(−)	-(−)
Positive rates (%)	1/60 (1.7)	4/60 (6.7)	2/60 (3.4)	2/60 (3.4)	0/60 (0)	2/60 (3.4)	1/60 (1.7)

Note: Data in the racket showed the result detected by uniplex PCR

## Discussion

Because of the development of mixed culture models, enhanced mobility of humans and animals, and environment pollution, viral infection in ducks is getting serious in recent years [1]. Among the popular viruses that infected ducks, AIV, NDV, NDPV, DHAV, DTMUV, FAdV, and DEV were very common, seriously endangering ducks’ health and causing great economic losses [5, 7, 10, 11, 28–30]. Although virus isolation is considered to be the gold standard for viral detection, it is unsuitable for clinical rapid detection [31]. Other methods, such as serological detection, immuno-electron microscopy, ELISA, and real-time PCR, have been applied for detecting these viruses [32–34]. However, rapid detection of the seven viruses simultaneously in ducks has not been reported. Therefore, we aim to develop a specific, sensitive, and rapid m-PCR method for clinical diagnosis of co-infection of duck virus. In establishing a successful multiplex PCR method, primer design is the key factor [35]. Mutual interference, such as mismatch and dimer [36], between different primers is common when different pairs of primers are mixed [37]. Within this experiment, a 5-plex PCR was first developed. When we enlarged the system into 7-plex PCR, with the addition of two pairs of new primers, interfering brands appeared, indicating that the original primers used in 5-plex PCR is unsuitable for 7-plex PCR. After careful optimization, seven pairs of primers for the viruses were found at last, with which seven viruses could be amplified separately. Moreover, these amplification products were consistent with the target gene fragments. The results proved that the m-PCR method could detect the seven viruses flexibly and that a usable m-PCR method has been established successfully.

Many other factors may affect the amplification efficiency. For example, even the smallest deviation of annealing temperature could lead to the nonspecific amplification [38]. Similarly, if Mg^2+^ in the reaction is extremely high, then the method lacks specificity, whereas if it is extremely low, then bad amplification can be expected [39]. Hence, by using the design of experiments, we optimized four pivotal parameters including annealing temperature, Mg^2+^ concentration, *Taq* DNA Polymerase concentration, and dNTP concentration that may influence the PCR [39]. A D-optimal design with 22 runs performed in one randomized batch (in duplicate measurements) was used for this optimization. The peak area was carefully investigated. In general, if a factor was considered critical, then the level, at which a factor gave the highest response, was used in the final experimental protocol [40]. In cases of conflicting results, the favorable level of a factor was decided in terms of the number of parameters from which the highest response was obtained. Therefore, a compromise was made to obtain the optimum parameters.

AIV, NDV, NDPV, DHAV, DTMUV, FAdV, DEV, and other viruses and bacteria that may infect ducks were utilized to evaluate the specificity of the m-PCR method. The result indicated that the m-PCR method produced neither cross reactions among these seven viruses nor nonspecific reactions with other common duck pathogens when all the DNA templates existed in the sample pool [41], thereby further demonstrating that the proposed primers were highly specific. The circulating NDPV is originated from the GPV lineage [30], thus the developed m-PCR method can also identify GPV at the same time. In addition, mix infection of different pathogens is common in clinical practice [42]. When ducks are infected with one virus, they are susceptible to others [9, 43], which may have a more powerful pathogenicity and bring about more serious economic losses [21]. Therefore, co-infection was performed to determine the practicality of the m-PCR method. The assay showed that the result of co-infection experiment was consistent with the grouping of seven viruses, which manifested that the m-PCR method could flexibly and specially detect several random virus combinations. The detection limit of the m-PCR method was 1 × 10^4^ copies/μL, which was higher than that of the uniplex PCR. The possible reason might be the mutual interference between multiple templates and primers [44]. However, considering its convenience and high throughput in sample analysis, the m-PCR method is more applicable. In addition, the m-PCR method showed highly consistent results with uniplex PCR, demonstrating the good accuracy of this method. The assay was performed to assess the repeatability and reproducibility of the method. This result suggested that the m-PCR method was highly reliable and stable [45].

The result of the field analysis with the developed m-PCR method was consistent with that of the uniplex PCR and standard PCR. Furthermore, all clinical positive samples were confirmed by sequencing. The samples collected from agricultural markets did not produce any specific band, and these positive samples were from different duck farms. Thus, the samples collected from the markets were healthy and safe. Furthermore, this result demonstrated that the m-PCR method was specific, sensitive, rapid, and practical in laboratory and clinical diagnoses.

## Conclusion

We established a multiplex PCR method with high specificity, good sensitivity, and reproducibility that could detect and differentiate seven major viruses causing duck diseases, including AIV, NDV, NDPV, DHAV, DTMUV, FAdV, and DEV. Thus, preventive measures can be implemented as early as possible to reduce economic losses.

## Methods

### Pathogen

DHAV (attenuated vaccine strain A66, purchased from Chengdu Tecbond Biological Products Co., Ltd. Cat. no. 220012214), DTMUV (attenuated vaccine strain WF100, purchased from QiLu Animal Health Products Co., Ltd. Cat. no. 1502522), and DEV (attenuated vaccine strain C-KCE, purchased from Guangxi Liyuan Biological Co., Ltd. Cat. no. 200352023) were isolated from the vaccine strain; AIV (H9N2), NDV, NDPV, goose parvovirus (GPV) and FAdV (serotype 4) were isolated from the clinical samples. Duck reovirus (DRV), Avian paramyxoviruses (APMV-4) *Escherichia coli* (wild-type, serotype O2), *Salmonella* (Typhimurium wild-type strain), *Riemerella anatipestifer* (wild-type strain, serotype 10), *Pasteurella multocida* (serotype ST129), and *Clostridium perfringens* (type A) were stored in our laboratory. F gene of APMV-8 were synthesized by Sangon Biotech.

### Nucleic acid extraction

The nucleic acid of the seven types of viruses was extracted using the Viral RNA/DNA Extraction Kit (Sangon Biotech, China) and dissolved with nuclease-free water. The RNAs of DHAV, DTMUV, AIV, and NDV were reverse-transcribed into cDNA by the Reverse Transcription Kit (Thermo Scientific, USA). The concentration and purity of each genome were determined by spectrophotometry (Thermo Scientific, USA). The DNA/cDNA was stored at − 20 °C.

### Primer design

The complete gene sequences of the DHAV, DTMUV, DEV, NDV, AIV, FAdV, and NDPV strains were acquired from the GenBank, and the conserved region of viral genes sequences was aligned by DNAMAN (LynnonBiosoft, USA).We designed seven pairs of specific primers for each virus by Primer Premier 5 (Premier, Canada), according to the results of the sequence alignment (Additional file 3: Figure S3). Primers that were listed in Table 1 (BLAST results of primers are shown in Additional file 4: Table S1) and synthesized by Sangon Biotech. The target genes include Matrix (M) gene for AIV, Fusion (F) gene for NDV, viral structural protein (VP3) gene for NDPV, viral structural protein (VP1) gene for DHAV, Envelope (E) gene for DTMUV, Hexon (H) gene for FAdV, and unique long region 2 (UL2) gene for DEV. These genes were the highly conserved region of these viruses (Table 1).

### Standard plasmid preparation

Seven recombinant plasmids were used as the positive controls. These were reconstructed in this study. Briefly, specific target fragments were amplified first with the primers (Table 1), and then these fragments were cloned into the pMD-18 T vector (TaKaRa, China) to obtain the recombinant plasmids pMD-AIV, pMD-NDV, pMD-NDPV, pMD-DHAV, pMD-DTMUV, pMD-FAdV, and pMD-DEV. The concentration of the recombinant plasmids was determined by NanoDrop-1000 (Thermo Fisher NanoDrop, USA), and the plasmid copy number was calculated using the following formula: copy number (copies/μL) = NA (copies/mol) × concentration (g/μL)/ MW (g/mol), where NA is Avogadro’s number, and MW is the base number times 340 [21].

### Uniplex PCR

The total volume of each reaction system was 25 μL, containing 2.5 μL 10 × *Taq* Buffer (Mg^2+^ free), 4 μL 25 mM Mgcl_2_, 0.75 μL dNTP Mix (10 mM each), 0.25 μL 5 U/μL *Taq* DNA Polymerase (Vazyme, China), 1 μL of the forward primer, 1 μL of the reverse primer, 1 μL of the single-virus template, and 14.5 μL of ddH_2_O. The PCR procedure was performed as follows: pre-denaturation at 95 °C for 5 min, followed by denaturation at 95 °C for 30 s, annealing at 57 °C for 30 s, extension at 72 °C for 40 s, 35 cycles, with a final extension at 72 °C for 10 min. The PCR products were analyzed by 1.5% agarose gel electrophoresis. Double distilled water was used as the blank control.

### Experimental design for multiplex PCR method

The m-PCR method was optimized using a D-optimal design consisting of 22 experiments. Four factors were considered including annealing temperatures (48 °C–62 °C), Mg^2+^ concentrations (1–6 mM), *Taq* DNA Polymerase concentrations (0.02–0.06 U/μL), and dNTP concentrations (0.08–0.48 mM). As a response, intensity of the PCR amplified bands (quantitated using the Image J (National Institutes of Health, Germany)) was used for statistical analysis [39, 46]. All analyses were performed using MODDE 12.1 software (Umetrics, Sweden). The relationship between the response Y and the variables X_i_, X_j_ was expressed as Y = β_0_ + β_i_X_i_ + β_j_X_j_ + β_ij_X_i_X_j_ + β_ii_X_i_^2^ + β_jj_X_j_^2^ +  … ε, where β_s_ represents the regression coefficients, and ε was the experimental error. The linear coefficients β_i_ and β_j_ were the quantitative effect of the respective variables. The cross coefficient β_ij_ measured the interaction between the variables, and the square terms of β_ii_X_i_^2^ and β_jj_X_j_^2^ described the non-linear effects on the response [47].

### Sensitivity and specificity of the m-PCR method

The mixed plasmids were diluted from 1 × 10^8^ copies/μL to 1 × 10^0^ by 10-fold gradient dilution and were used to detect the sensitivity of the m-PCR method. The specificity of m-PCR was tested using other common duck viruses and bacteria (including DRV, APMV-4, APMV-8, DHAV-3, *Escherichia coli*, *Salmonella*, *Riemerella anatipestifer*, *Pasteurella multocida, and Clostridium perfringens*).

### Repeatability and reproducibility of the m-PCR method

The repeatability of the method was evaluated at three concentrations on the same day, whereas the reproducibility study was performed at three concentration levels on three successive days. The concentrations of seven recombinant plasmids were 1 × 10^4^, 1 × 10^5^, and 1 × 10^6^ copies/μL [44]. In addition, the repeatability and reproducibility of the uniplex PCR were carried out with the plasmid concentration of 1 × 10^2^, 1 × 10^3^, 1 × 10^4^, and 1 × 10^5^ copies/μL.

### Co-infection model and clinical specimen detection

Co-infection analysis was designed to determine the practicality of the m-PCR method [45]. Furthermore, 60 clinical samples (40 tissue samples and 20 throat swabs) were collected from the duck farms and live poultry markets in Jiangsu province (These samples collection were permitted by the owner of the animals and suffering were minimized to these animals). The established m-PCR method was used for PCR amplification. Results were further confirmed by uniplex PCR and standard or published PCR methods [1, 48–52]. Then, the positive products of PCR amplification were sequenced to confirm the detection results.

## Additional files

Additional file 1: **Figure S1.** Repeatability and reproducibility of the uniplex PCR and m-PCR methods. The plasmid concentration for reproducibility analysis of the uniplex PCR for FAdV (A) and DHAV (B) was set at 10^3^, 10^4^, and 10^5^ copies/μL, and that for DEV (C), DTUMV (D), NDV (E), AIV (F), and NDPV (G) were set at 10^2^, 10^3^, and 10^4^copies/μL. The plasmid concentration for reproducibility analysis of the m-PCR (H) was set at 10^4^, 10^5^, and 10^6^ copies/μL. (JPG 229 kb)

Additional file 2: **Figure S2.** Co-infection analysis by m-PCR method. Detection result of co-infection of two viruses with plasmid concentration of 1 × 10^4^ copies/μL. Lanes 1 and 9, positive control; Lanes 2 and 8, NDV + DTMUV; Lanes 3 and 7, AIV + DEV; Lanes 4 and 6, DHAV+NDPV; Lane 5, AIV + FAdV; M, DL600 marker; NC, negative control. (JPG 155 kb)

Additional file 3: **Figure S3.** Alignment results of the seven viruses’ primers. (DOCX 791 kb)

Additional file 4: **Table S1.** BLAST results of AIV forward primer and NDV forward primer. (DOCX 15 kb)

## Abbreviations

AIV: Avian influenza virus
*C. perfringens*: *Clostridium perfringens*
ddH_2_O: Double distilled water
DEV: Duck enteritis virus
DHAV: Duck hepatitis A virus
dNTP: Deoxy-ribonucleotide triphosphate
DRV: Duck reovirus
DTMUV: Duck Tembusu virus
E: Envelope
*E.coli*: *Escherichia coli*
ELISA: Enzyme-linked immune sorbent assay
F: Fusion
FAdV: Fowl adenovirus
FAO: Food and Agriculture Organization
GPV: Goose parvovirus
H: Hexon
LFA: Lateral flow assay
M: DL600 marker
M: Matrix
Mg^2+^: Magnesian ion
m-PCR: Multiplex PCR
MW: Base number times
NA: Avogadro’s number
NC: Negative control
NDPV: Novel duck parvovirus
NDV: Newcastle disease virus
*P. multocida*: *Pasteurella multocida*
PCR: Polymerase Chain Reaction
RA: *Riemerella anatipestifer*
SE: *Salmonella*
UL2: Unique long region 2
VP1: Viral structural protein
VP3: Viral structural protein

## References

[1] Wang Y, Zhu S, Hong W, Wang A, Zuo W (2017). A multiplex PCR for detection of six viruses in ducks. J Virol Methods.

[2] Zhang YF, Xie ZX, Xie LJ, Deng XW, Xie ZQ, Luo SS, Huang L, Huang JL, Zeng TT (2015). GeXP analyzer-based multiplex reverse-transcription PCR assay for the simultaneous detection and differentiation of eleven duck viruses. BMC Microbiol.

[3] Haider N, Sturm-Ramirez K, Khan SU, Rahman MZ, Sarkar S, Poh MK, Shivaprasad HL, Kalam MA, Paul SK, Karmakar PC (2017). Unusually High Mortality in Waterfowl Caused by Highly Pathogenic Avian Influenza A(H5N1) in Bangladesh. Transbound Emerg Dis.

[4] Kwon YK, Joh SJ, Kim MC, Sung HW, Lee YJ, Choi JG, Lee EK, Kim JH (2005). Highly pathogenic avian influenza (H5N1) in the commercial domestic ducks of South Korea. Avian Pathol.

[5] Dai Y, Cheng X, Liu M, Shen X, Li J, Yu S, Zou J, Ding C (2014). Experimental infection of duck origin virulent Newcastle disease virus strain in ducks. BMC Vet Res.

[6] Li C, Li Q, Chen Z, Liu G (2016). Novel duck parvovirus identified in Cherry Valley ducks (Anas platyrhynchos domesticus), China. Infect Genet Evol.

[7] Lin SL, Cong RC, Zhang RH, Chen JH, Xia LL, Xie ZJ, Wang Y, Zhu YL, Jiang SJ (2016). Circulation and in vivo distribution of duck hepatitis A virus types 1 and 3 in infected ducklings. Arch Virol.

[8] Erfan AM, Selim AA, Moursi MK, Nasef SA, Abdelwhab EM (2015). Epidemiology and molecular characterisation of duck hepatitis A virus from different duck breeds in Egypt. Vet Microbiol.

[9] Shen HQ, Lin WC, Wang ZX, Zhang K, Yan ZQ, Zhou QF, Qin JP, Xie QM, Bi YZ, Chen F (2016). Pathogenicity and genetic characterization of a duck Tembusu virus associated with egg-dropping in Muscovy ducks. Virus Res.

[10] Niczyporuk JS, Wozniakowski G, Samorek-Salamonowicz E (2015). Application of cross-priming amplification (CPA) for detection of fowl adenovirus (FAdV) strains. Arch Virol.

[11] Gao X, Jia R, Wang M, Yang Q, Chen S, Liu M, Yin Z, Cheng A (2017). Duck enteritis virus (DEV) UL54 protein, a novel partner, interacts with DEV UL24 protein. Virol J.

[12] Niu X, Chen H, Yang J, Yu X, Ti J, Wang A, Diao Y (2016). Development of a TaqMan-based real-time PCR assay for the detection of Novel GPV. J Virol Methods.

[13] Bergeron HC, Glas PS, Schumann KR (2017). Diagnostic specificity of the African swine fever virus antibody detection enzyme-linked immunosorbent assay in feral and domestic pigs in the United States. Transbound Emerg Dis.

[14] Deng J, Li S, Hong J, Ji Y, Zhou Y (2013). Investigation on subcellular localization of Rice stripe virus in its vector small brown planthopper by electron microscopy. Virol J.

[15] Killian ML (2014). Hemagglutination assay for influenza virus. Methods Mol Biol.

[16] Zhao K, He W, Bi J, Zhang X, Zhang D, Huang H, Zhang Y, Song D, Gao F (2016). Development of a lateral flow immunochromatographic assay for the rapid diagnosis of Orf virus infections. J Virol Methods.

[17] Perkasa A, Yudhaputri F, Haryanto S, Hayati RF, Ma'roef CN, Antonjaya U, Yohan B, Myint KS, Ledermann JP, Rosenberg R (2016). Isolation of Zika Virus from Febrile Patient, Indonesia. Emerg Infect Dis.

[18] Luo Y, Atim SA, Shao L, Ayebazibwe C, Sun Y, Liu Y, Ji S, Meng XY, Li S, Li Y (2017). Development of an updated PCR assay for detection of African swine fever virus. Arch Virol.

[19] Xu XG, Chen GD, Huang Y, Ding L, Li ZC, Chang CD, Wang CY, Tong DW, Liu HJ (2012). Development of multiplex PCR for simultaneous detection of six swine DNA and RNA viruses. J Virol Methods.

[20] Junlong L, Li Y, Liu A, Guan G, Xie J, Yin H, Luo J (2015). Development of a multiplex PCR assay for detection and discrimination of Theileria annulata and Theileria sergenti in cattle. Parasitol Res.

[21] Gao Q, Yun B, Wang Q, Jiang L, Zhu H, Gao Y, Qin L, Wang Y, Qi X, Gao H (2014). Development and application of a multiplex PCR method for rapid differential detection of subgroup A, B, and J avian leukosis viruses. J Clin Microbiol.

[22] Smith KL, Dunstan RA (1993). PCR detection of cytomegalovirus: a review. Br J Haematol.

[23] Mirmajlessi SM, Destefanis M, Gottsberger RA, Mänd M, Loit E (2015). PCR-based specific techniques used for detecting the most important pathogens on strawberry: a systematic review. Systematic Reviews.

[24] Chen LL, Xu Q, Zhang RH, Yang L, Li JX, Xie ZJ, Zhu YL, Jiang SJ, Si XK (2013). Improved duplex RT-PCR assay for differential diagnosis of mixed infection of duck hepatitis A virus type 1 and type 3 in ducklings. J Virol Methods.

[25] Hao L, Xie J, Chen S, Wang S, Gong Z, Ling KS, Guo L, Fan Z, Zhou T (2016). A multiple RT-PCR assay for simultaneous detection and differentiation of latent viruses and apscarviroids in apple trees. J Virol Methods.

[26] Almeida S, Dorneles EMS, Diniz C, Abreu V, Sousa C, Alves J, Carneiro A, Bagano P, Spier S, Barh D (2017). Quadruplex PCR assay for identification of Corynebacterium pseudotuberculosis differentiating biovar Ovis and Equi. BMC Vet Res.

[27] Moustacas VS, Silva TM, Costa LF, Xavier MN, Carvalho CA, Costa EA, Paixao TA, Santos RL (2013). Species-specific multiplex PCR for the diagnosis of Brucella ovis, Actinobacillus seminis, and Histophilus somni infection in rams. BMC Vet Res.

[28] Ramey AM, Kim Torchetti M, Poulson RL, Carter D, Reeves AB, Link P, Walther P, Lebarbenchon C, Stallknecht DE (2016). Evidence for wild waterfowl origin of H7N3 influenza A virus detected in captive-reared New Jersey pheasants. Arch Virol.

[29] Xu Y, Gong P, Wielstra B, Si Y (2016). Southward autumn migration of waterfowl facilitates cross-continental transmission of the highly pathogenic avian influenza H5N1 virus. Sci Rep.

[30] Fan W, Sun Z, Shen T, Xu D, Huang K, Zhou J, Song S, Yan L (2017). Analysis of Evolutionary Processes of Species Jump in Waterfowl Parvovirus. Front Microbiol.

[31] Chen HT, Zhang J, Sun DH, Zhang JL, Cai XP, Liu XT, Ding YZ, Ma LN, Yang SH, Jin L (2008). Rapid discrimination of H5 and H9 subtypes of avian influenza viruses and Newcastle disease virus by multiplex RT-PCR. Vet Res Commun.

[32] Jiang T, Liu J, Deng YQ, Su JL, Xu LJ, Liu ZH, Li XF, Yu XD, Zhu SY, Gao GF (2012). Development of RT-LAMP and real-time RT-PCR assays for the rapid detection of the new duck Tembusu-like BYD virus. Arch Virol.

[33] Zeynalova S, Guliyev F, Vatani M, Abbasov B (2015). Biosurveillance of avian influenza and Newcastle disease viruses in the Barda region of Azerbaijan using real time RT-PCR and hemagglutination inhibition. Front Microbiol.

[34] Huang Q, Yue H, Zhang B, Nie P, Tang C (2012). Development of a real-time quantitative PCR for detecting duck hepatitis a virus genotype C. J Clin Microbiol.

[35] Alvarez-Fernandez R (2013). Explanatory chapter: PCR primer design. Methods Enzymol.

[36] Shen Z, Qu W, Wang W, Lu Y, Wu Y, Li Z, Hang X, Wang X, Zhao D, Zhang C (2010). MPprimer: a program for reliable multiplex PCR primer design. BMC Bioinformatics.

[37] Balenger SL, McClure CJ, Hill GE (2012). Primer design and transcript quantification of a highly multiplexed RT-PCR for a nonmodel avian species. Mol Ecol Resour.

[38] Rychlik W, Spencer WJ, Rhoads RE (1990). Optimization of the annealing temperature for DNA amplification in vitro. Nucleic Acids Res.

[39] Obradovic J, Jurisic V, Tosic N, Mrdjanovic J, Perin B, Pavlovic S, Djordjevic N (2013). Optimization of PCR conditions for amplification of GC-Rich EGFR promoter sequence. J Clin Lab Anal.

[40] Liu Y, Liu Q, Hesketh J, Huang D, Gan F, Hao S, Tang S, Guo Y, Huang K (2018). Protective effects of selenium-glutathione-enriched probiotics on CCl4-induced liver fibrosis. J Nutr Biochem.

[41] Lee JH, Seo HJ, Park JY, Kim SH, Cho YS, Kim YJ, Cho IS, Jeoung HY (2015). Detection and differentiation of Schmallenberg, Akabane and Aino viruses by one-step multiplex reverse-transcriptase quantitative PCR assay. BMC Vet Res.

[42] Pantin-Jackwood MJ, Costa-Hurtado M, Miller PJ, Afonso CL, Spackman E, Kapczynski DR, Shepherd E, Smith D, Swayne DE (2015). Experimental co-infections of domestic ducks with a virulent Newcastle disease virus and low or highly pathogenic avian influenza viruses. Vet Microbiol.

[43] Zhang X, Jiang S, Wu J, Zhao Q, Sun Y, Kong Y, Li X, Yao M, Chai T (2009). An investigation of duck circovirus and co-infection in Cherry Valley ducks in Shandong Province, China. Vet Microbiol.

[44] Zeng Z, Liu Z, Wang W, Tang D, Liang H, Liu Z (2014). Establishment and application of a multiplex PCR for rapid and simultaneous detection of six viruses in swine. J Virol Methods.

[45] Liu J, Yao L, Zhai F, Chen Y, Lei J, Bi Z, Hu J, Xiao Q, Song S, Yan L (2018). Development and application of a triplex real-time PCR assay for the simultaneous detection of avian influenza virus subtype H5, H7 and H9. J Virol Methods.

[46] García LT, Cristancho LM, Vera EP, Begambre O (2015). A New Multiplex-PCR for Urinary Tract Pathogens Detection Using Primer Design Based on an Evolutionary Computation Method. J Microbiol Biotechnol.

[47] Zhu R, Zhao Z, Wang J, Bai B, Wu A, Yan L, Song S (2015). A simple sample pretreatment method for multi-mycotoxin determination in eggs by liquid chromatography tandem mass spectrometry. J Chromatogr A.

[48] Hu Q, Zhu D, Ma G, Cheng A, Wang M, Chen S, Jia R, Liu M, Sun K, Yang Q (2016). A one-step duplex rRT-PCR assay for the simultaneous detection of duck hepatitis A virus genotypes 1 and 3. J Virol Methods.

[49] Spackman E, Senne DA, Myers TJ, Bulaga LL, Garber LP, Perdue ML, Lohman K, Daum LT, Suarez DL (2002). Development of a real-time reverse transcriptase PCR assay for type A influenza virus and the avian H5 and H7 hemagglutinin subtypes. J Clin Microbiol.

[50] Steer PA, Kirkpatrick NC, O'Rourke D, Noormohammadi AH (2009). Classification of fowl adenovirus serotypes by use of high-resolution melting-curve analysis of the hexon gene region. J Clin Microbiol.

[51] Wise MG, Suarez DL, Seal BS, Pedersen JC, Senne DA, King DJ, Kapczynski DR, Spackman E (2004). Development of a real-time reverse-transcription PCR for detection of newcastle disease virus RNA in clinical samples. J Clin Microbiol.

[52] Xie L, Xie Z, Huang L, Wang S, Huang J, Zhang Y, Zeng T, Luo S (2017). A polymerase chain reaction assay for detection of virulent and attenuated strains of duck plague virus. J Virol Methods.

