# Supplementary figures and images for: Development and application of multiplex PCR method for simultaneous detection of seven viruses in ducks
Source: BMC Vet Res. 2019 Apr 1;15:103. doi: 10.1186/s12917-019-1820-1 (PMC6444421; doi:10.1186/s12917-019-1820-1)

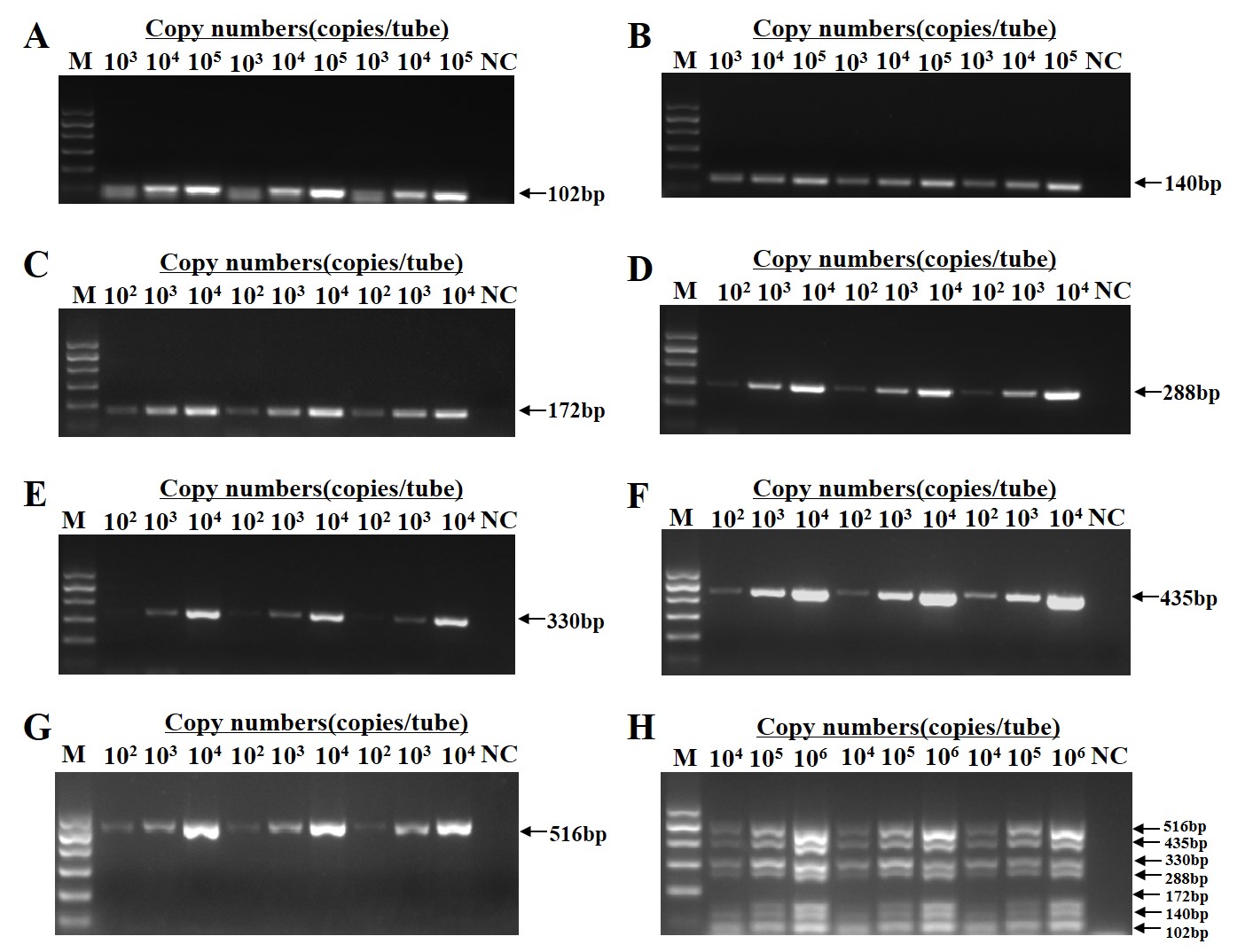

Supplement: Supplementary file 1 — Figure S1. Repeatability and reproducibility of the uniplex PCR and m-PCR methods. The plasmid concentration for reproducibility analysis of the uniplex PCR for FAdV (A) and DHAV (B) was set at 103, 104, and 105 copies/μL, and that for DEV (C), DTUMV (D), NDV (E), AIV (F), and NDPV (G) were set at 102, 103, and 104copies/μL. The plasmid concentration for reproducibility analysis of the m-PCR (H) was set at 104, 105, and 106 copies/μL. (JPG 229 kb) [file 12917_2019_1820_MOESM1_ESM.jpg]

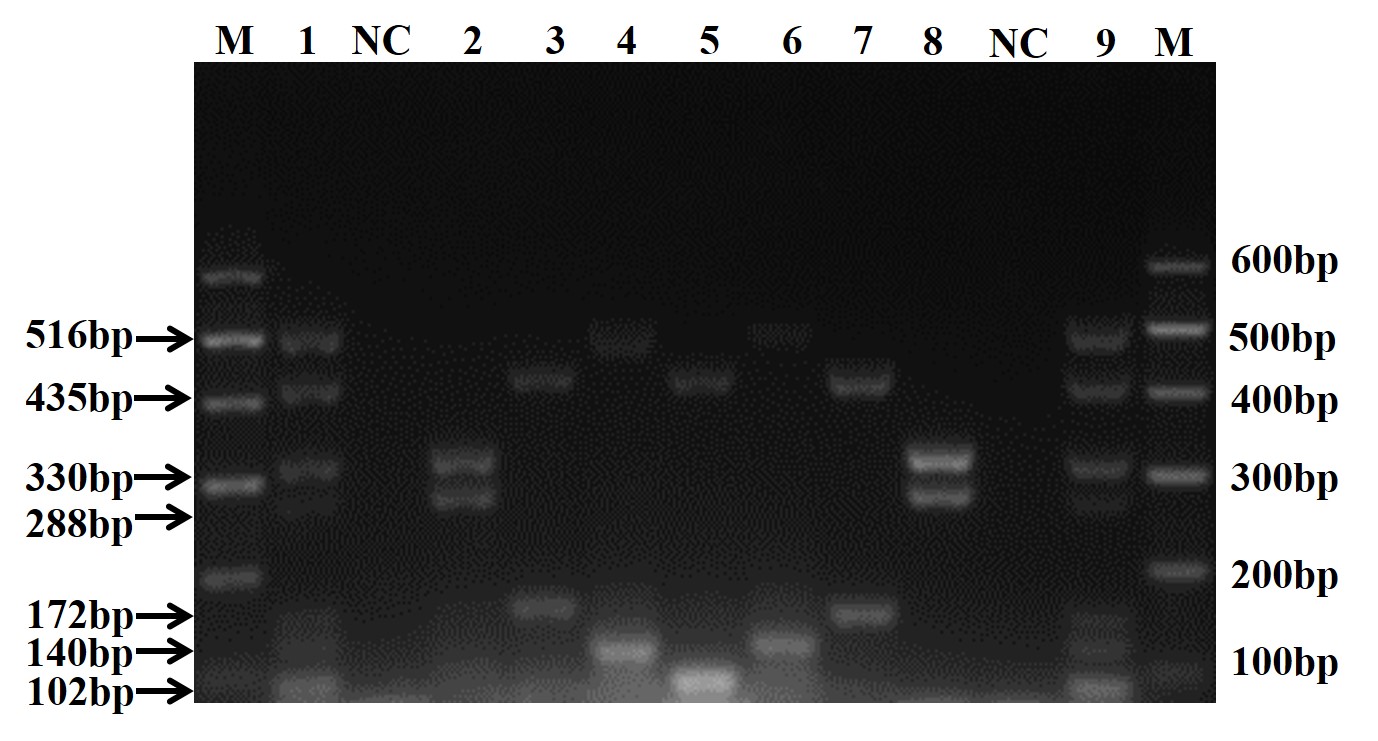

Supplement: Supplementary file 2 — Figure S2. Co-infection analysis by m-PCR method. Detection result of co-infection of two viruses with plasmid concentration of 1 × 10^4^ copies/μL. Lanes 1 and 9, positive control; Lanes 2 and 8, NDV + DTMUV; Lanes 3 and 7, AIV + DEV; Lanes 4 and 6, DHAV+NDPV; Lane 5, AIV + FAdV; M, DL600 marker; NC, negative control. (JPG 155 kb) [file 12917_2019_1820_MOESM2_ESM.jpg]
