# Supplementary material for: Development and application of multiplex PCR method for simultaneous detection of seven viruses in ducks
Source: BMC Vet Res. 2019 Apr 1;15:103. doi: 10.1186/s12917-019-1820-1 (PMC6444421; doi:10.1186/s12917-019-1820-1)

**Alignment results of the seven viruses’ primers.**

1. Alignment result of FAdV F primer.


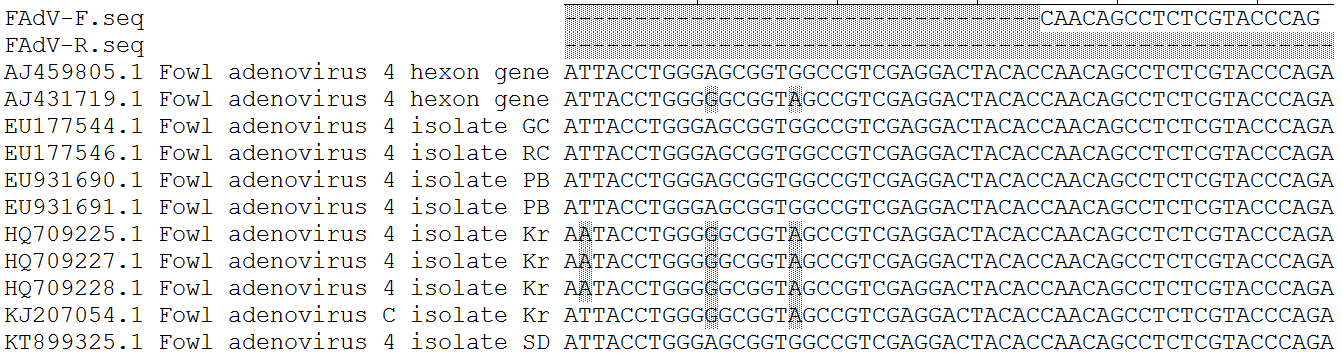


Alignment result of FAdV R primer. (Reverse complementary sequence)


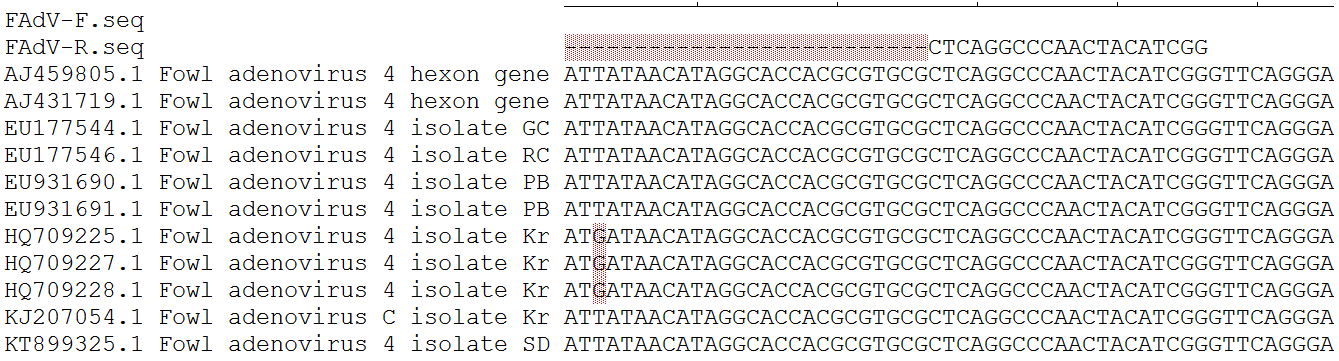


1. Alignment result of DHAV F primer.


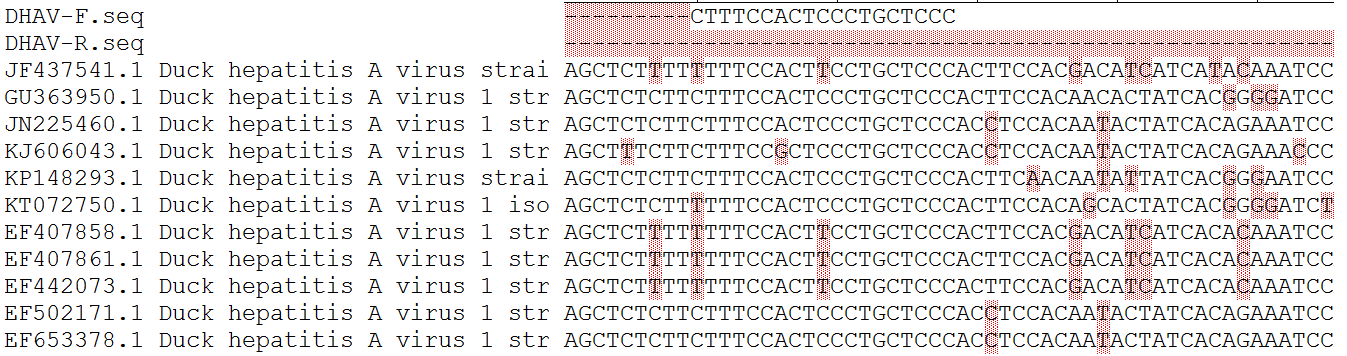


Alignment result of DHAV R primer. (Reverse complementary sequence)


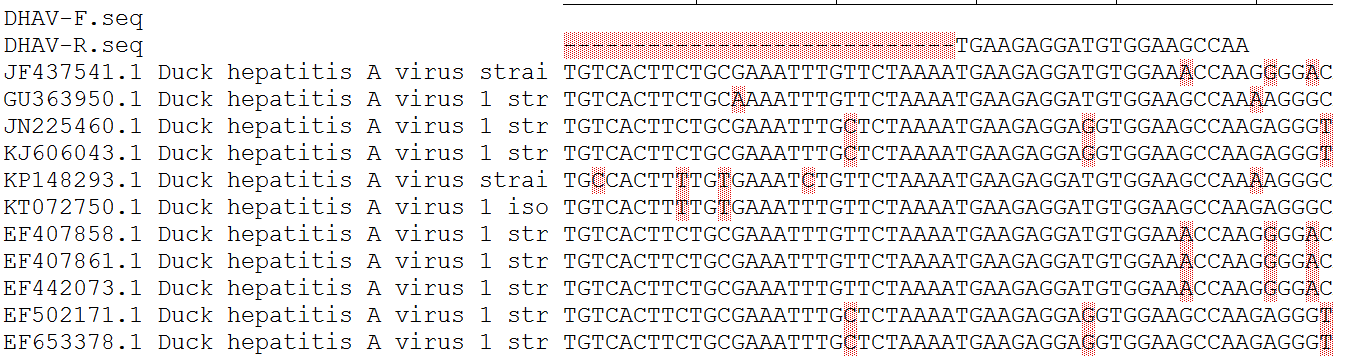


1. Alignment result of DEV F primer.


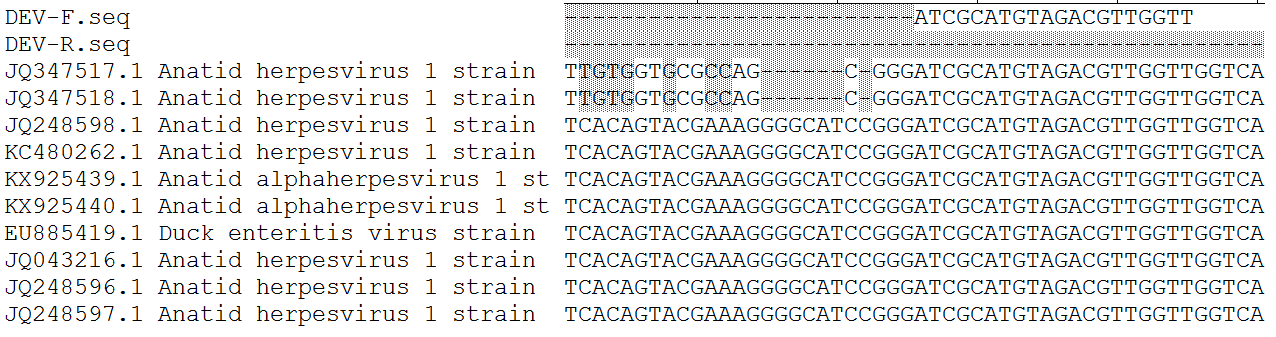


Alignment result of DEV R primer. (Reverse complementary sequence)


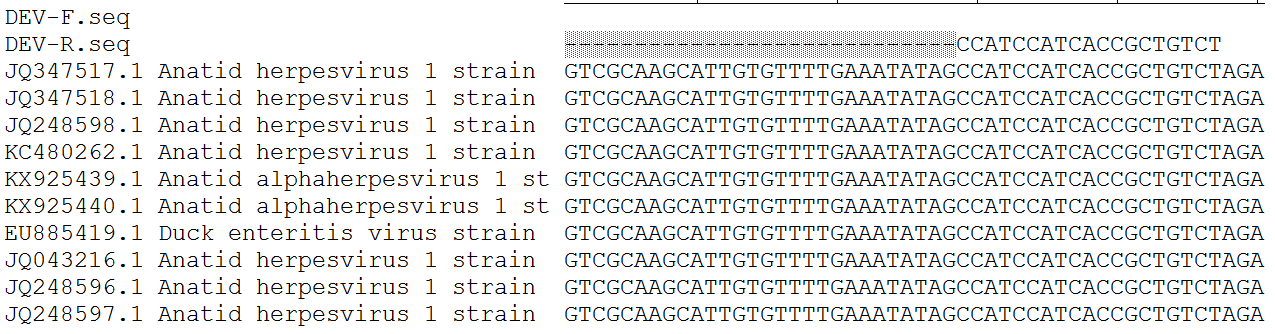


1. Alignment result of DTMUV F primer.


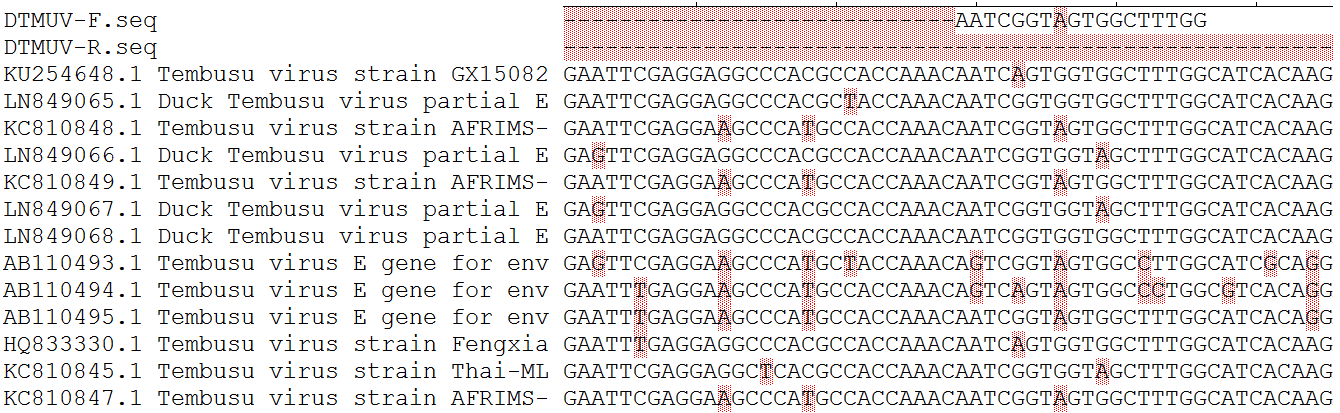


Alignment result of DTMUV R primer. (Reverse complementary sequence)


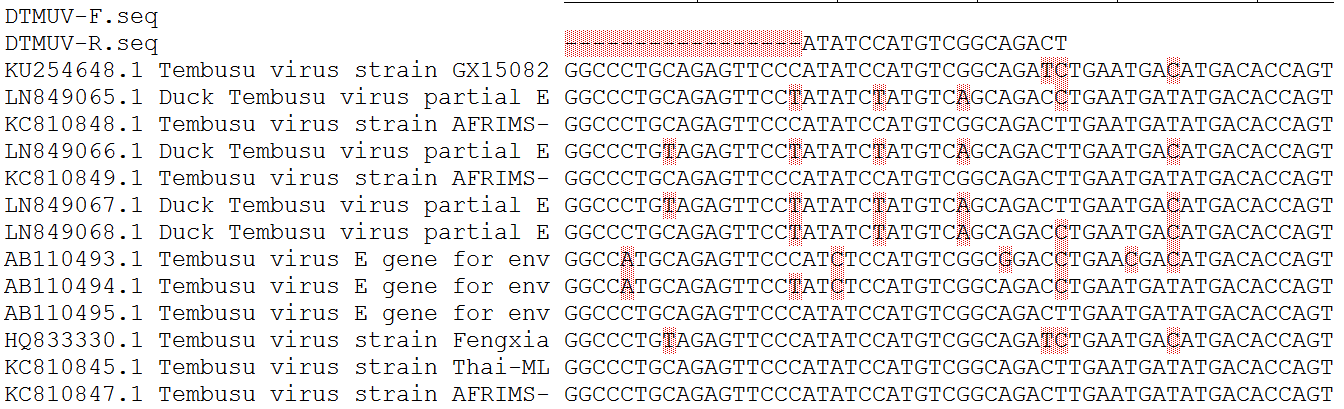


1. Alignment result of NDV F primer.


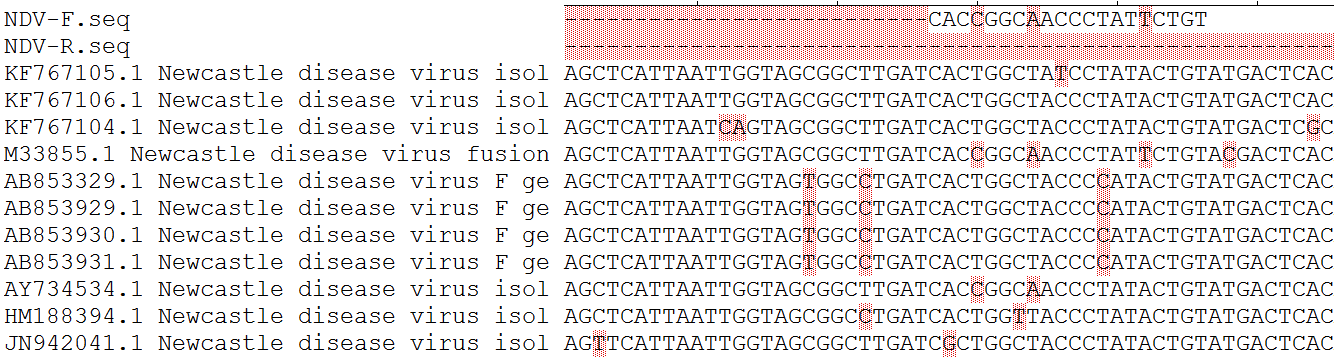


Alignment result of NDV R primer. (Reverse complementary sequence)


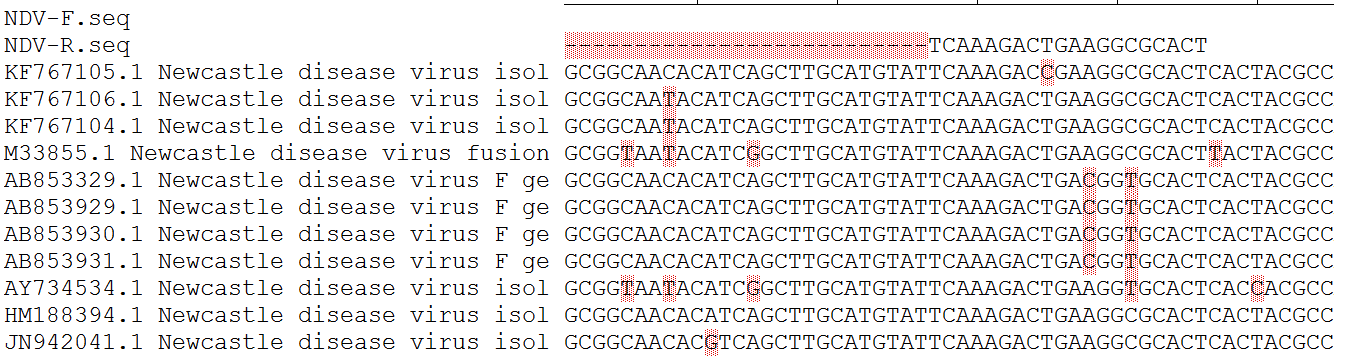


1. Alignment result of AIV F primer.


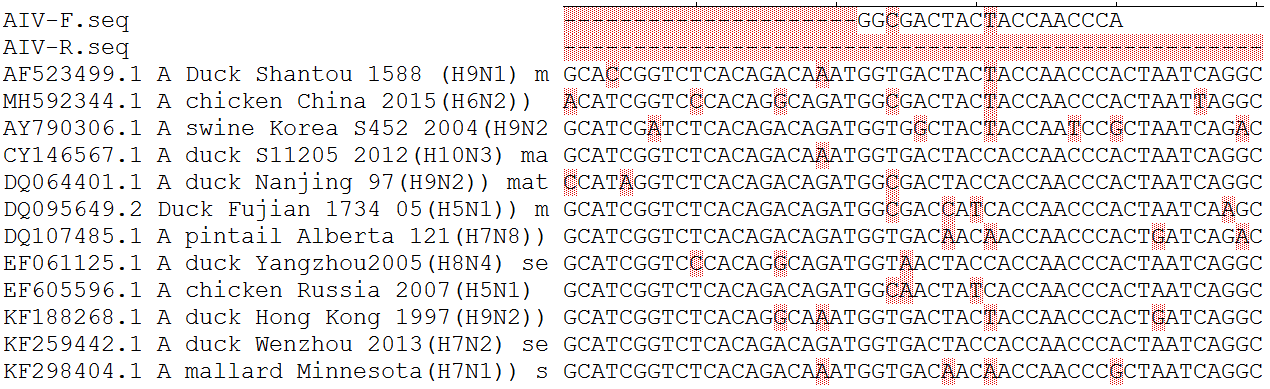


Alignment result of AIV R primer. (Reverse complementary sequence)


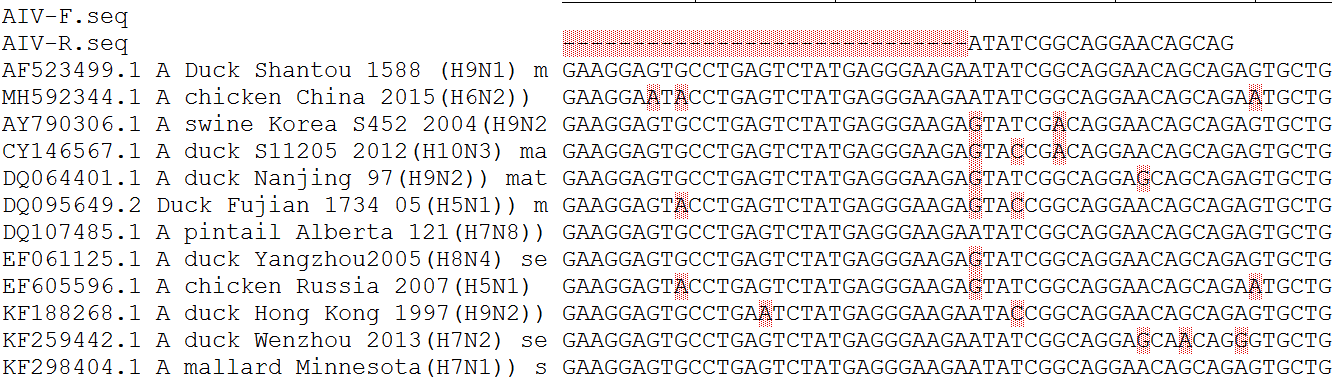


1. Alignment result of NDPV F primer.


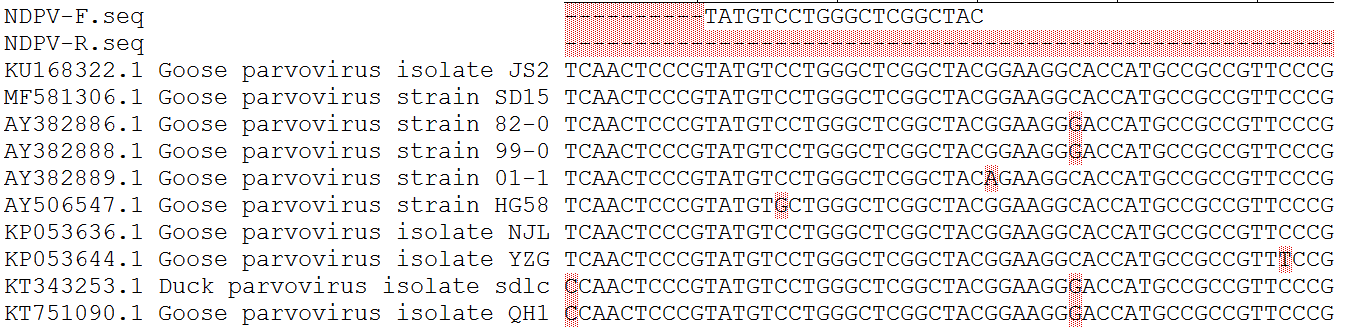


Alignment result of NDPV R primer. (Reverse complementary sequence)


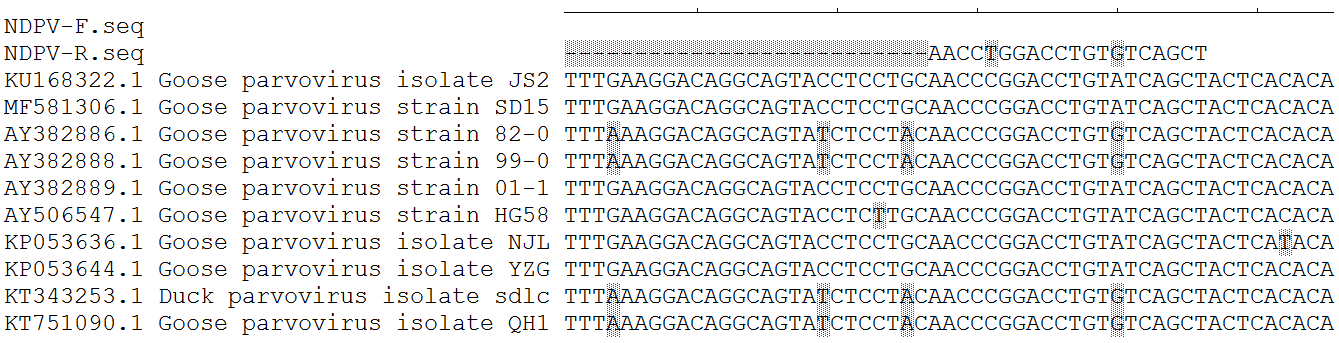

Supplement: Supplementary file 3 — Figure S3. Alignment results of the seven viruses’ primers. (DOCX 791 kb) [file 12917_2019_1820_MOESM3_ESM.docx]
