# Supplementary material for: Development and application of multiplex PCR method for simultaneous detection of seven viruses in ducks
Source: BMC Vet Res. 2019 Apr 1;15:103. doi: 10.1186/s12917-019-1820-1 (PMC6444421; doi:10.1186/s12917-019-1820-1)
Supplement: Supplementary file 4 — Table S1. BLAST results of AIV forward primer and NDV forward primer. (DOCX 15 kb) [file 12917_2019_1820_MOESM4_ESM.docx]

**Table S1.** The BLAST results of AIV forward primer and NDV forward primer.

| Name of virus | Name of primers | The results of BLAST | Identity ratios |
| --- | --- | --- | --- |
| AIV | F primer | [Influenza A virus](https://blast.ncbi.nlm.nih.gov/Blast.cgi?CMD=Get&RID=411RKEGJ01R&FORMAT_OBJECT=TaxBlast&DESCRIPTIONS=100&PROGRAM=blastn&QUERY_INDEX=0) H5 | 100% |
|  |  | [Influenza A virus](https://blast.ncbi.nlm.nih.gov/Blast.cgi?CMD=Get&RID=411RKEGJ01R&FORMAT_OBJECT=TaxBlast&DESCRIPTIONS=100&PROGRAM=blastn&QUERY_INDEX=0) H6 | 100% |
|  |  | [Influenza A virus](https://blast.ncbi.nlm.nih.gov/Blast.cgi?CMD=Get&RID=411RKEGJ01R&FORMAT_OBJECT=TaxBlast&DESCRIPTIONS=100&PROGRAM=blastn&QUERY_INDEX=0) H7 | 100% |
|  |  | [Influenza A virus](https://blast.ncbi.nlm.nih.gov/Blast.cgi?CMD=Get&RID=411RKEGJ01R&FORMAT_OBJECT=TaxBlast&DESCRIPTIONS=100&PROGRAM=blastn&QUERY_INDEX=0) H9 | 100% |
| NDV | F primer | [Newcastle disease virus](https://blast.ncbi.nlm.nih.gov/Blast.cgi#alnHdr_1095952453) | 100% |
